# Nuclear receptor subfamily 4 group A member 2 inhibits activation of ERK signaling and cell growth in response to β-adrenergic stimulation in adult rat cardiomyocytes

**DOI:** 10.1152/ajpcell.00526.2018

**Published:** 2019-06-12

**Authors:** Sadia Ashraf, Yassmin K. Hegazy, Romain Harmancey

**Affiliations:** ^1^Department of Physiology and Biophysics, University of Mississippi Medical Center, Jackson, Mississippi; ^2^Mississippi Center for Obesity Research, University of Mississippi Medical Center, Jackson, Mississippi; ^3^Mississippi Center for Heart Research, University of Mississippi Medical Center, Jackson, Mississippi

**Keywords:** adrenergic signaling, heart failure, hypertrophy, nuclear receptor, MAPK

## Abstract

Sustained elevation of sympathetic activity is an important contributor to pathological cardiac hypertrophy, ventricular arrhythmias, and left ventricular contractile dysfunction in chronic heart failure. The orphan nuclear receptor NR4A2 is an immediate early-response gene activated in the heart under β-adrenergic stimulation. The goal of this study was to identify the transcriptional remodeling events induced by increased NR4A2 expression in cardiomyocytes and their impact on the physiological response of those cells to sustained β-adrenergic stimulation. Treatment of adult rat ventricular myocytes with isoproterenol induced a rapid (<4 h) increase in NR4A2 levels that was accompanied by a transient (<24 h) increase in nuclear localization of the transcription factor. Adenovirus-mediated overexpression of NR4A2 to similar levels modulated the expression of genes linked to adrenoceptor signaling, calcium signaling, cell growth and proliferation and counteracted the increase in protein synthesis rate and cell surface area mediated by chronic isoproterenol stimulation. Consistent with those findings, NR4A2 overexpression also blocked the phosphorylative activation of growth-related kinases ERK1/2, Akt, and p70 S6 kinase. Prominent among the transcriptional changes induced by NR4A2 was the upregulation of the dual-specificity phosphatases DUSP2 and DUSP14, two known inhibitors of ERK1/2. Pretreatment of NR4A2-overexpressing cardiomyocytes with the DUSP inhibitor BCI [(*E*)-2-benzylidene-3-(cyclohexylamino)-2,3-dihydro-1*H*-inden-1-one] prevented the inhibition of ERK1/2 following isoproterenol stimulation. In conclusion, our results suggest that NR4A2 acts as a novel negative feedback regulator of the β-adrenergic receptor-mediated growth response in cardiomyocytes and this at least partly through DUSP-mediated inhibition of ERK1/2 signaling.

## INTRODUCTION

Heart failure with reduced left ventricular ejection fraction is a serious health condition with a 50% mortality rate within five years after diagnosis (30). As the number of Americans diagnosed with heart failure is expected to increase by nearly 40% during the next 15 years, the costs of managing the illness will skyrocket, rising from $31 billion in 2012 to $70 billion by 2030 (16). Hyperactivity of the sympathoadrenergic system plays a central role in the transition from compensated to decompensated heart failure. Although increased β-adrenergic signaling initially helps maintain the pumping function of the heart, chronic activation eventually leads to inflammation, pathological hypertrophy, impaired calcium homeostasis, and ultimately death of cardiomyocytes (22, 41). Consequently, use of β-blockers has become a cornerstone therapy for the treatment of patients with chronic heart failure (4, 25). However, lack of specificity from current β-adrenergic blocking agents precludes preservation of the protective and prosurvival pathways linked to increased sympathetic nervous system activity (3). Thus, a better understanding of β-adrenergic signaling and of its regulation in cardiomyocytes may provide novel therapeutic insights for improved management of heart failure, increased survival of patients, and decreased burden on the American healthcare system.

The NR4A subfamily of nuclear receptors is composed of the three members NR4A1/NUR77, NR4A2/NURR1, and NR4A3/NOR-1. All three NR4A members are expressed in cardiomyocytes (27). In the mouse heart, the expression of the NR4A receptors rapidly increases in a transient manner following injection of the β-adrenergic receptor agonist isoproterenol (31). NR4A1 and NR4A3 have recently emerged as key players in cardiac stress response to various pathological stimuli such as pressure overload and myocardial infarction (26). Several studies have highlighted contrasting roles for both receptors in the regulation of cardiac remodeling in response to chronic β-adrenergic receptor activation, with NR4A1 acting as an antihypertrophic factor while NR4A3 appears to be prohypertrophic (11, 27, 42). We (8) previously reported strong induction of NR4A2 in the rat heart subjected to surgically induced hypothermic ischemic arrest and reperfusion. However, despite its well-established effects in the regulation of energy homeostasis, inflammation, growth, and survival in multiple tissues, the impact of NR4A2 on cardiac physiology remains largely unknown (26).

In this work, we tested the hypothesis that NR4A2 is induced in cardiomyocytes following acute stimulation of β-adrenergic signaling. We further hypothesized that chronic activation of NR4A2 modulates the hypertrophic response of cardiomyocytes to sustained β-adrenergic stimulation. We used adenovirus-mediated overexpression of the full-length form of human NR4A2 to determine the transcriptional remodeling events mediated by activation of the orphan nuclear receptor in adult rat ventricular myocytes (ARVMs). Transcriptome profiling by RNA sequencing was combined with targeted analysis of the identified cellular signal transduction pathways under baseline conditions and following isoproterenol stimulation.

## MATERIALS AND METHODS

### 

#### Reagents and vectors.

Type 2 and type 4 collagenases were purchased from Worthington Biochemical. The chemically defined lipid concentrate was obtained from GIBCO. The laminin mouse protein was purchased from Invitrogen. The dual-specificity protein phosphatase (DUSP) inhibitor (*E*)-2-benzylidene-3-(cyclohexylamino)-2,3-dihydro-1*H*-inden-1-one (BCI) was purchased from EMD Millipore. All other reagents were obtained from Sigma-Aldrich. The human NR4A2 adenovirus (Ad-h-NR4A2; cat. no. ADV-217057) and enhanced green fluorescent protein (eGFP) adenovirus (Ad-GFP; cat. no. 1060) were generated, amplified, purified, and titrated by Vector Biolabs.

#### Animals.

Male Sprague-Dawley rats were obtained from Envigo and housed in the Center for Comparative Research animal facilities of the University of Mississippi Medical Center (UMMC). Rats were kept on a 12:12-h light-dark cycle and fed a standard laboratory rodent diet (Teklad 8640). Rats weighing between 250 and 350 g were euthanized under isoflurane anesthesia (4–5% for induction and 2–3% for maintenance) for heart tissue recovery. The animal use protocol was conducted in accordance with the National Institutes of Health’s *Guide for the Care and Use of Laboratory Animals* and was approved by the UMMC Institutional Animal Care and Use Committee.

#### Cell cultures.

H9c2 (1, 2) rat cardiac myoblasts were obtained directly from ATCC along with a certificate of analysis (ATCC cat. no. CRL-1446, RRID:CVCL_0286). Consequently, cell line authentication and mycoplasma contamination tests were not performed in our laboratory. H9c2 cells were grown in DMEM containing 584 mg/L l-glutamine, 110 mg/L sodium pyruvate, 4.5 g/L d-glucose and supplemented with 10% (vol/vol) fetal bovine serum, 100 U/mL penicillin, and 100 µg/mL streptomycin. Adult rat ventricular myocytes (ARVMs) were isolated according to a modified version of the method developed by Ackers-Johnson and colleagues (1). In brief, rats anesthetized with 2–3% inhaled isoflurane were intravenously injected with 200 United States Pharmacopeia (USP) units of heparin, and their hearts were subsequently removed and immediately transferred into ice-cold EDTA buffer. Following aortic cannulation, the hearts were retrogradely perfused first with syringes filled with 20 mL of ice-cold EDTA buffer to wash them free of blood and then with 40 mL of ice-cold perfusion buffer, and finally with 40 mL of recirculating collagenase buffer prewarmed to 38°C. After proceeding with mechanical dissociation of heart tissue, cell separation by gravity settlement, and calcium reintroduction, ARVMs were plated at a density of 5,000–55,000 cells/cm^2^ in plating medium (medium 199, 5% (vol/vol) fetal bovine serum, 10 mmol/L 2,3-butanedione monoxime (BDM), 100 U/mL penicillin, and 100 µg/mL streptomycin) on laminin-coated tissue culture dishes. One hour after plating, the plating medium was replaced with culture medium (medium M199, 0.1% (wt/vol) bovine serum albumin, 1× insulin-transferrin-selenium, 10 mmol/L BDM, 1× chemically defined lipid concentrate, 100 U/mL penicillin, and 100 µg/mL streptomycin).

#### Cell treatments.

The protocol used for overexpression of NR4A2 in ARVMs and subsequent analysis of the effects on cell growth and hypertrophy are described in Supplemental Fig. S1 (Supplemental data: https://doi.org/10.6084/m9.figshare.7492751). Briefly, cardiomyocytes were transduced with either Ad-GFP or Ad-h-NR4A2 [50 multiplicity of infection (MOI)] at the time of plating medium replacement with culture medium. An MOI of 50 led to 100% transduction efficiency. At 48 h posttransduction, cells were processed for determination of NR4A2-mediated transcriptional reprogramming by RNA sequencing or further treated with isoproterenol (10 μmol/L) to determine the impact of NR4A2 overexpression on β-adrenergic-mediated intracellular signaling at 10 min poststimulation, changes in rates of protein synthesis at 24 h poststimulation, and hypertrophy at 48 h poststimulation.

#### Real-time PCR analysis of mRNA levels.

ARVMs were seeded onto laminin-coated six-well plates. Total RNA was isolated from cultured cells using TRIzol Reagent (Invitrogen) and treated for residual DNA contamination with DNA-free (Invitrogen). One-half microgram of DNase-treated RNA was reverse transcribed by use of SuperScript III reverse transcriptase (Invitrogen). Relative quantification of target mRNA levels was performed with self-designed primers and TaqMan probes on a ViiA 7 real-time PCR system (Applied Biosystems). Data were normalized using the geometric mean of housekeeping genes RNA18S, GAPDH, and peptidylprolyl isomerase A. A reverse transcriptase minus reaction served as a negative control for each gene quantified. Sequences for primers and probes are provided in Supplemental Table S1 (https://doi.org/10.6084/m9.figshare.7492751).

#### Immunofluorescence.

Immunofluorescence experiments were carried out following the *Guidelines for Authors and Reviewers on Antibody Use in Physiology Studies* (5). Cells were grown in 24-well culture plates on laminin-coated coverslips. Following experimental treatments, the cells were washed three times with PBS and fixed for 15 min at room temperature with 4% (wt/vol) paraformaldehyde solution in PBS. Cells were then washed three times with PBS and incubated for 1 h at room temperature in blocking solution containing 10% (vol/vol) horse serum, 1% (wt/vol) bovine serum albumin (BSA), and 0.1% (vol/vol) Triton X-100 in PBS. After blocking was completed, cells were incubated overnight at 4°C with primary antibody, washed three times in PBS, and then further incubated for 1 h at room temperature with DyLight secondary antibody (Vector Laboratories). Actin cytoskeleton in H9c2 cardiac myoblasts was visualized using Phalloidin-iFluor 594 Reagent (Abcam) following the manufacturer’s instructions. The coverslips were mounted on glass slides using VECTASHIELD Antifade Mounting Medium with DAPI (Vector Laboratories). Fluorescence signal was analyzed with an Olympus IX 81 inverted fluorescence microscope and a Nikon confocal C1+ microscope. A detailed list of primary antibodies used in immunofluorescence analyses is provided in Supplemental Table S2 (https://doi.org/10.6084/m9.figshare.7492751).

#### Western blotting.

ARVMs were seeded on laminin-coated 100-mm tissue culture dishes. Following drug treatments, ARVMs were scraped in ice-old PBS, centrifuged at 1,200 *g* for 5 min at 4°C, and resuspended in M-PER Mammalian Protein Extraction Reagent (Thermo Scientific) supplemented with phosphatase inhibitor cocktails 2 and 3 (Sigma-Aldrich) and Complete Mini protease inhibitor cocktail (Roche Applied Science). After being gently shaken for 10 min, cells were disrupted with an ultrasonic probe sonicator using 3 cycles at 25% amplitude (10 s ON/60 s OFF) and the lysates were centrifuged at 14,000 *g* for 15 min to remove cell debris. Protein concentration of the supernatant was determined by bicinchoninic acid assay. Proteins were separated by polyacrylamide gel electrophoresis and transferred to 0.45-µm pore size polyvinylidene difluoride membranes. Membranes were blocked for 1 h with 5% milk in 1× Tris-buffered saline, and 0.4% Tween 20 at room temperature and incubated overnight at 4°C with primary antibody. Protein detection was carried out using horseradish peroxidase-conjugated secondary antibody and chemiluminescence. Densitometric analyses were performed with ImageJ 1.48v. A detailed list of primary antibodies used in Western blot analyses is provided in Supplemental Table S2. Use of antibody and immunoblot analysis complied with the *Guidelines for Authors and Reviewers on Antibody Use in Physiology Studies* (5).

#### [^3^H]leucine incorporation assay.

ARVMs were seeded on laminin-coated six-well plates. Cells were treated with vehicle or isoproterenol in the presence of [^3^H]leucine (1 mCi/mL, 149 Ci mmol^−1^ specific activity, PerkinElmer) for 24 h. After treatment, cells were washed three times with PBS, solubilized in 1 mL of lysis buffer (10% SDS, 20% glycerol, 100 mM Tris, pH 6.8), and diluted in nine-tenth volume of scintillation fluid, and the radioactivity was measured in a scintillation counter.

#### RNA sequencing.

RNA was extracted using the PureLink RNA Mini Kit (Ambion) according to manufacturer instructions and assessed for quality control parameters of minimum concentration and fidelity (i.e., 18S and 28S bands, RQI >8). cDNA libraries were developed using the TruSeq Stranded mRNA Library Prep Kit (Set-A-indexes), evaluated by Qubit fluorometer (Invitrogen), and assessed for quality and size using the Bio-Rad Experion System as done previously (29). Samples were pooled into a single library (*n* = 12 pooled samples per library) and sequenced using the NextSeq 500 High Output Kit (300 cycles, paired end 100 bp) on the Illumina NextSeq 500 platform. The run generated 136.4 Gb at QC30 = 87.5% with 670 million or 55 million reads passing filter. Sequenced reads were assessed for quality using the Illumina Basespace Cloud Computing Platform, and FASTQ sequence files were used to align reads to the rat reference genome [*Rattus norvegicus*/Rn5 (Refseq)] using the RNA-Seq Alignment Application (using STAR aligner). On average, 54 million reads (or >95% reads per sample) were mapped to the reference genome. Differential expression was determined using Cufflinks Assembly & DE workflow (v.2.1.0). After removal of duplicates and genes with one or more missing data point, differentially expressed genes were filtered on the basis of a ≥1.5-fold difference between fragments per kilobase of transcript per million mapped reads (FPKM) values and *q* < 0.05. RNA sequencing data were deposited in the Gene Expression Omnibus database under accession no. GSE122911.

#### Statistical analyses.

Statistical analyses complied with the report of minimum details needed per recommendation established in the *Statistical Considerations in Reporting Cardiovascular Research* (20). Data are expressed as means ± SE. Comparisons between two groups were performed using unpaired, two-tailed Student’s *t*-test. Comparisons between multiple experimental groups were made by one-way analysis of variance with Newman-Keuls post hoc test. A *P* value < 0.05 was considered statistically significant. All the analyses were performed with GraphPad Prism (version 7).

## RESULTS

### 

#### Generation of a highly pure population of adult rat ventricular myocytes.

Proper identification of physiological effects of NR4A2 on cardiomyocytes first required accurate and reproducible isolation of healthy ARVMs from other cardiac cell populations. Isolated ARVMs displayed typical rod-shaped structure for more than 96 h in culture (Supplemental Fig. S2*A*; https://doi.org/10.6084/m9.figshare.7492751). Real-time PCR analysis confirmed high mRNA levels for myocyte markers Myh6, Myh7, and Tnnt2 in the ARVMs fraction. Conversely, mRNA levels of fibroblast markers Vim, Col1a1, and Col1a2 were 1,000 to 2,000 times lower than the levels measured in the nonmyocyte cell fraction (Supplemental Fig. S2*B*). Probing of cultured cells by immunofluorescence further confirmed that ARVM cultures, unlike the nonmyocyte cell fraction, were devoid of protein markers specific for endothelial cells (RECA-1), fibroblasts (Vimentin), smooth muscle cells (Sm22-α), and macrophages (CD68; Supplemental Fig. S2*C*). The Lindsey group (29) recently used RNA sequencing for comparative cell marker selection and confirmation of successful isolation and purification of a single cell type from cardiac tissue. Using a similar approach, we found that average FPKM values for cardiomyocyte-specific markers varied between 184 and 2,368 in our ARVM cultures, while FPKM values for nonmyocyte cell markers ranged only between 0 and 6 (Supplemental Fig. S2*D*). Taken together, the results indicated that highly pure ARVM populations were used for the present experiments.

#### β-Adrenergic receptor stimulation induces transient expression of NR4A2 in cardiomyocytes.

**Fig. 1.**
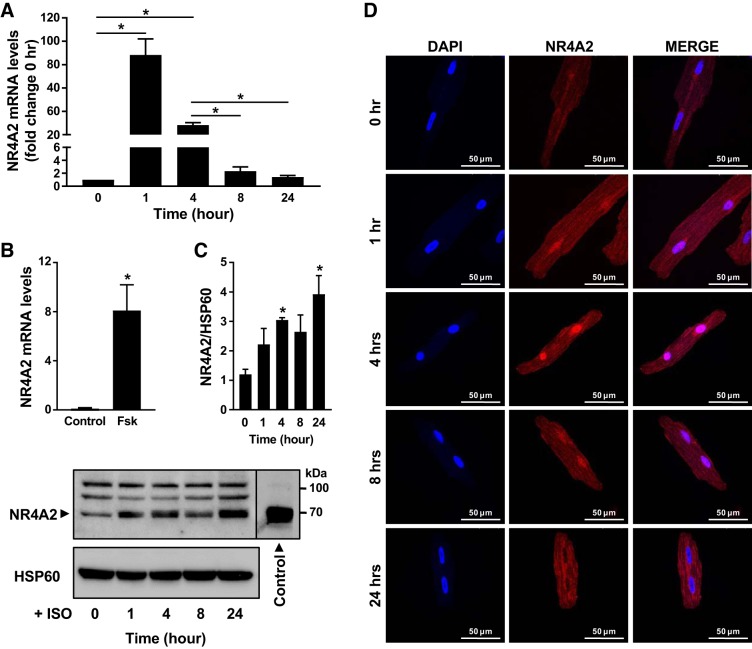
cAMP-mediated β-adrenergic signaling stimulates expression and nuclear localization of nuclear receptor subfamily 4 group A (NR4A2) in adult cardiomyocytes. *A*: time course of NR4A2 mRNA expression level in response to 10 µmol/L isoproterenol (ISO). *B*: NR4A2 mRNA expression levels in cells treated for 1 h with 10 µmol/L forskolin (Fsk). HSP60, heat shock protein 60. *C*: time course of NR4A2 protein expression level in response to 10 µmol/L isoproterenol. A representative immunoblot is presented. The control [adult rat ventricular myocytes (ARVMs) transduced with adenoviral (Ad)-h-NR4A2] was loaded on the same gel as samples but developed separately to prevent overexposure. *D*: time course of NR4A2 immunolocalization in response to 10 µmol/L isoproterenol. Pairwise comparison was performed with unpaired Student’s *t*-test. Interaction between multiple groups was determined by one-way ANOVA, including Newman-Keuls post hoc analysis when significant interaction occurred. Data are means ± SE of 3–5 independent experiments using ARVMs isolated from different rats. *P < 0.05.

Transcript levels of all three members of the NR4A subgroup of orphan nuclear receptors have been shown to rapidly and transiently increase in vivo in the mouse heart in response to treatment with the β-adrenergic agonist isoproterenol. However, the relative contributions of the different cardiac cell types to this increase remained unknown (31). In cultured ARVMs, stimulation with 10 μmol/L isoproterenol for 1 h led to an 88-fold increase in NR4A2 mRNA levels. Transcript levels of NR4A2 then steadily decreased, down by 68% at 4 h and back to near-baseline levels at 24 h, after initial stimulation (Fig. 1*A*). A similar increase in NR4A2 mRNA level was observed when ARVMs were stimulated with forskolin for 1 h, suggesting that β-adrenergic stimulation of NR4A2 transcription is mediated by a cAMP-dependent signaling pathway (Fig. 1*B*). Isoproterenol similarly induced transient expression of NR4A2 in H9c2 cardiomyoblasts, although to a much lesser extent (Supplemental Fig. S3*A*; https://doi.org/10.6084/m9.figshare.7492751). At the protein level, NR4A2 expression increased in ARVMs for the next 24 h following isoproterenol treatment (Fig. 1*C*). Increased NR4A2 expression was accompanied by increased NR4A2 immunoreactivity in cardiomyocyte nuclei. However, nuclear localization was only transient and peaked at 4 h after the beginning of stimulation before returning to baseline levels by 24 h posttreatment (Fig. 1*D*). In summary, the results suggest that cardiomyocytes are a major source of NR4A2 expression in the rodent heart and that NR4A2 contributes to transcriptional remodeling of those cells in response to adrenergic stimulation.

#### NR4A2 modulates expression of genes encoding proteins involved in adrenergic signaling, cell growth, and calcium signaling.

**Fig. 2.**
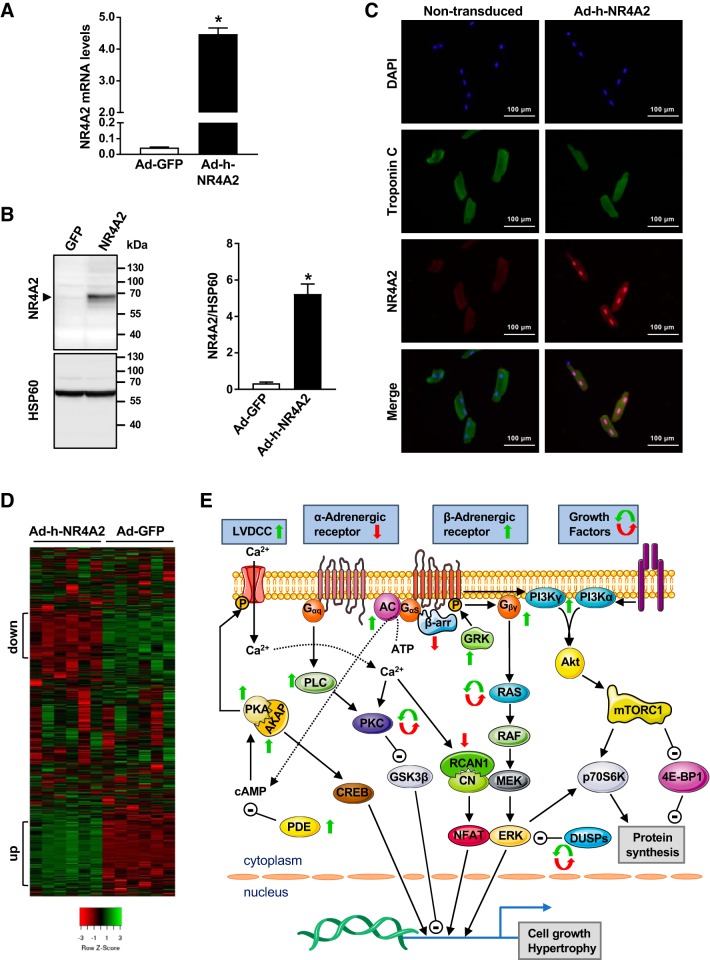
RNA-Seq analysis identifies nuclear receptor subfamily 4 group A (NR4A2) as a transcriptional modulator of growth regulatory pathways in adult cardiomyocytes. *A*: comparison of NR4A2 total mRNA levels in adenovirus encoding green fluorescent protein (Ad-GFP) control and Ad-h-NR4A2 transduced cells. *B*: comparison of NR4A2 total protein levels in Ad-GFP control and Ad-h-NR4A2-transduced cells. Representative immunoblot is presented. *C*: immunolocalization of NR4A2 in nontransduced control and Ad-h-NR4A2-transduced cells. Troponin C was used as marker for cardiac myocytes. Pairwise comparison was performed with unpaired Student’s *t*-test. Data are means ± SE of 4–5 independent experiments using adult rat ventricular myocytes (ARVMs) isolated from different rats. *P < 0.05. *D*: heat map representation of 3,021 genes that passed filtering criteria. The 2 clusters of genes differentially regulated (down- and upregulated, respectively) in NR4A2-overexpressing cells compared with the GFP-expressing control cells are indicated. *E*: selective representation of cell growth and hypertrophy signaling networks containing effectors regulated at gene level in response to NR4A2 overexpression. Upward green and downward red arrows represent increased and decreased expression levels, respectively. Circular arrows indicate changes in isoform expression. Data are based on analysis of 6 independent experiments using ARVMs isolated from different rats. LVDCC, L-type voltage-dependent Ca^2+^ channel; β-arr, β-arrestin; GRK, G protein-coupled receptor kinase; AKAP, A-kinase anchoring protein; RCAN1, regulator of calcineurin 1; mTORC1, mammalian target of rapamycin complex 1; PDE, phosphodiesterase; CREB, cAMP response element-binding protein; GSK-3β, glycogen synthase kinase-3β; DUSP, dual-specificity phosphatase; PI3K, phosphoinositide 3-kinase; 4E-BP1, eukaryotic initiation factor 4E-binding protein-1.

To determine the transcriptional remodeling events induced by increased NR4A2 expression in adult cardiomyocytes, ARVMs were transduced with an adenovirus containing the full-length open reading frame of human NR4A2 under transcriptional control of the cytomegalovirus promoter (Ad-h-NR4A2). When compared with cells transduced with a control recombinant adenovirus encoding green fluorescent protein (Ad-GFP), transduction with Ad-h-NR4A2 increased total NR4A2 mRNA and protein levels 100- and 15-fold, respectively (Fig. 2, *A* and *B*). The overexpressed protein localized mainly in the nucleus, as visualized by immunofluorescence (Fig. 2*C*). Therefore, transgenic expression of NR4A2 in cardiomyocytes reproduced the short-term effects of isoproterenol stimulation on induction and localization of the endogenously expressed transcription factor.

The impact of NR4A2 overexpression on the transcriptome of ARVMs was then investigated with RNA sequencing. Among the 3,021 genes that passed the filtering criteria, 850 genes were differentially regulated compared with the Ad-GFP-expressing control cells (fold change ≥1.5). Among the differentially regulated genes, 500 were upregulated and 350 were downregulated (Supplemental Table S3; https://doi.org/10.6084/m9.figshare.7492751). The majority of differentially regulated genes varied consistently among all biological replicates and clustered into two distinct sets on the heatmap representation of gene expression data (Fig. 2*D*). Targeted pathway analysis of the two gene clusters revealed that NR4A2 overexpression regulates the expression of genes involved in adrenoceptor signaling, growth and proliferation, and calcium signaling (Table 1). Signaling pathways known to regulate cardiac hypertrophy were particularly represented and included the adenylate cyclase-phosphodiesterase-protein kinase A (PKA) axis, the α-adrenoceptor-heterotrimeric protein G_q_-protein kinase C (PKC) signaling, the calcium-calmodulin pathway, the Ras-Raf-MAPK pathway, and the phosphoinositide-Akt kinase signaling (Fig. 2*E*). All together, the transcriptomic data revealed that NR4A2 modulates gene expression networks related to growth and hypertrophy of ARVMs in response to adrenergic stimulation.

**Table 1.** Subset of genes differentially regulated by NR4A2 in ARVMs that are related to adrenoceptor signaling, growth and proliferation, and calcium signaling

Gene Symbol	Description	Accession No.	Regulation by NR4A2	Fold Change
Adrenergic receptor signaling				
* Adrb2*	Adrenoceptor β2	NM_012492	↑	3.1
* Adra1a*	Adrenoceptor α1A	NM_017191	↓	0.4
* Adra1b*	Adrenoceptor α1B	NM_016991	↓	0.5
* Adra1d*	Adrenoceptor α1D	NM_024483	↓	0.4
* Arrdc3*	Arrestin domain-containing protein-3	NM_001007797	↓	0.5
* Grk5*	G protein-coupled receptor kinase-5	NM_030829	↑	2.9
* Gng3*	G protein subunit-γ3	NM_053658	↑	2.9
* Rgs16*	Regulator of G protein signaling 16	NM_001077589	↑	5.4
* Adcy6*	Adenylate cyclase-6	NM_012821	↑	3.8
* Prkar2b*	Protein kinase cAMP-dependent type II regulatory subunit-β	NM_001030020	↑	3.3
* Akap2*	A-kinase-anchoring protein-2	NM_001011974	↑	4.5
* Pde2a*	Phosphodiesterase-2A	NM_031079	↑	3.5
* Pde4d*	Phosphodiesterase-4D	NM_017032	↑	2.2
* Pde10a*	Phosphodiesterase-10A	NM_022236	↑	6.4
* Plch1*	Phospholipase Cη1	NM_001191707	↑	24.0
* Prkca*	Protein kinase Cα	NM_001105713	↑	2.2
* Prkcq*	Protein kinase Cθ	NM_001276721	↓	0.2
Growth and proliferation				
* Fgf1*	Fibroblast growth factor 1	NM_012846	↓	0.3
* Fgf9*	Fibroblast growth factor 9	NM_012952	↓	0.4
* Fgfr3*	Fibroblast growth factor receptor 3	NM_053429	↑	4.6
* Pik3r3*	Phosphoinositide-3-kinase regulatory subunit 3	NM_022213	↑	11.2
* Pik3ip1*	Phosphoinositide-3-kinase interacting protein-1	NM_001017453	↓	0.3
* Rab7b*	Ras-related protein Rab-7b	NM_001109328	↓	0.1
* Rab33b*	Ras-related protein Rab-33b	NM_001108944	↑	2.9
* Rasl11b*	Ras-like family 11 member b	NM_001002830	↑	4.3
* Rap1b*	RAP1B, member of RAS oncogene family	NM_134346	↑	2.1
* Rac2*	Rac family small GTPase 2	NM_001008384	↓	0.3
* Rhov*	Ras homolog family member V	NM_138542	↑	3.7
* Arf2*	ADP ribosylation factor 2	NM_024150	↑	2.2
* Rap1gds1*	Rap1 GTPase-GDP dissociation stimulator 1	NM_001107728	↑	4.1
* Rapgef5*	Rap guanine nucleotide exchange factor 5	NM_001047915	↑	6.9
* Rassf5*	Ras association domain family member 5	NM_019365	↑	2.4
* Rgl1*	Ral guanine nucleotide dissociation stimulator-like 1	NM_001105957	↓	0.5
* Map3k8*	Mitogen-activated protein kinase kinase kinase-8	NM_053847	↑	2.9
* Dusp2*	Dual-specificity phosphatase-2	NM_001012089	↑	7.7
* Dusp14*	Dual-specificity phosphatase-14	NM_001270835	↑	7.1
* Dusp15*	Dual-specificity phosphatase-15	NM_001244784	↓	0.4
Calcium signaling				
* Cacnb3*	Calcium voltage-gated channel auxiliary subunit-β3	NM_012828	↑	4.5
* Cacna2d3*	Calcium voltage-gated channel auxiliary subunit-α2δ3	NM_175595	↑	3.0
* Slc8a1*	Sodium/calcium exchanger 1	NM_019268	↑	2.4
* Rcan1*	Regulator of calcineurin 1	NM_153724	↓	0.2

Fold change ratio to green fluorescent protein-expressing control cells based on the mean from six independent experiments using adult rat ventricular myocytes (ARVMs) isolated from different rats. NR4A2, nuclear receptor subfamily 4 group A.

#### Chronic NR4A2 overexpression blocks isoproterenol-mediated protein synthesis and growth of cardiomyocytes.

**Fig. 3.**
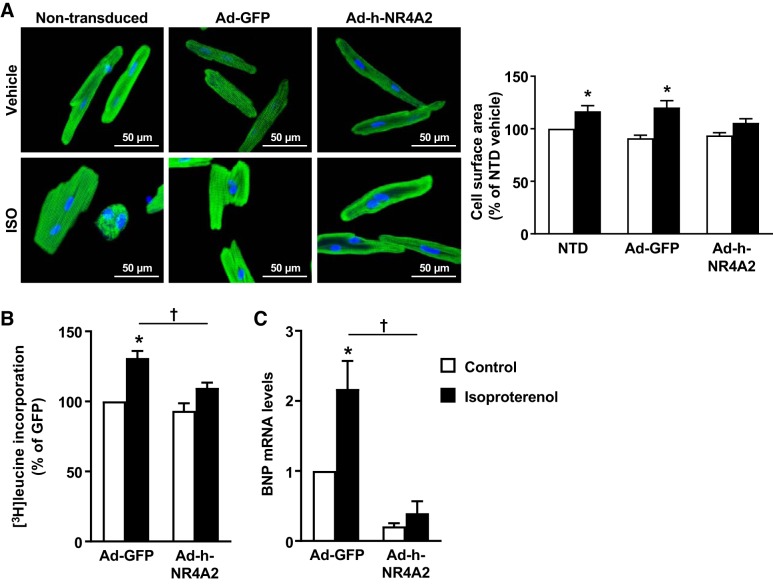
Nuclear receptor subfamily 4 group A (NR4A2) blocks the growth response of adult cardiomyocytes to isoproterenol (ISO). *A*: immunofluorescence visualization (troponin C) and quantification of adult rat ventricular myocyte (ARVM) surface area in response to 48-h treatment with 10 µmol/L isoproterenol in nontransduced (NTD), green fluorescent protein (GFP)-expressing, and NR4A2-overexpressing cells. Measurements performed on average of 266 cells per condition. *B*: measurement of cellular protein synthesis rates by incorporation of [^3^H]leucine over a 24-h period following treatment with 10 µmol/L isoproterenol. *C*: brain natriuretic peptide (BNP) mRNA expression levels in GFP-expressing control and NR4A2-overexpressing ARVMs following 48-h treatment with 10 µmol/L isoproterenol. Interaction between multiple groups was determined by one-way ANOVA, including a Newman-Keuls post hoc analysis when significant interaction occurred. Data are means ± SE of 3–5 independent experiments using ARVMs isolated from different rats. *P < 0.05 vs. control; †*P* < 0.05.

Long-term β-adrenergic receptor stimulation with isoproterenol is a potent inducer of protein synthesis and growth in isolated adult cardiomyocytes (38). In our hands, treatment of ARVMs with 10 µmol/L isoproterenol for 48 h led, on average, to a 20% increase in surface area for control, nontransduced, or Ad-GFP-expressing cells. This increase in cell surface area was inhibited by chronic overexpression of NR4A2 (Fig. 3*A*). The antigrowth effect of NR4A2 overexpression was even more dramatic in H9c2 cardiomyoblasts, where the 40–60% increase in cell size induced by isoproterenol was completely reversed following transduction with Ad-h-NR4A2 (Supplemental Fig. S3*B*). Consistent with the changes in cell surface area, the isoproterenol-mediated increase in rates of protein synthesis was also significantly decreased by NR4A2 overexpression (Fig. 3*B*). Interestingly, NR4A2 overexpression also potently inhibited expression of the cardiac hypertrophy marker B-type natriuretic peptide (BNP) in both vehicle- and isoproterenol-treated ARVMs (Fig. 3*C*). Thus, sustained NR4A2 overexpression blocks the growth response of cardiomyocytes to chronic β-adrenergic receptor stimulation.

#### NR4A2 inhibits ERK1/2 and Akt activation in response to isoproterenol stimulation.

**Fig. 4.**
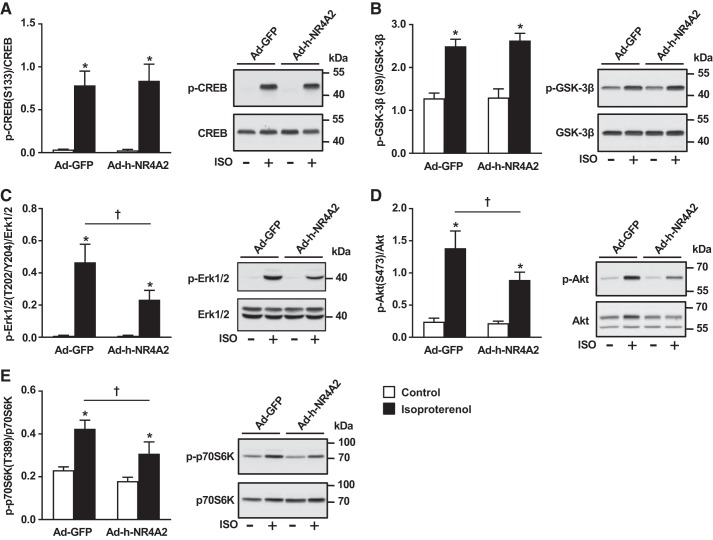
Nuclear receptor subfamily 4 group A (NR4A2) inhibits isoproterenol (ISO)-mediated ERK, Akt, and p70 S6 kinase (S6K) phosphorylation in adult cardiomyocytes. Phosphorylation (p) status of cAMP response element-binding protein (CREB; *A*), glycogen synthase kinase-3β (GSK-3β; *B*), extracellular signal-regulated kinases (ERK1/2; *C*), Akt kinase (*D*), and p70S6K kinase (*E*) was quantified by immunoblotting in green fluorescent protein (GFP)-expressing control and NR4A2-overexpressing cells at baseline and after 10-min stimulation with 10 µmol/L isoproterenol. Interaction between multiple groups was determined by one-way ANOVA, including Newman-Keuls post hoc analysis when significant interaction occurred. Data are means ± SE of 3–4 independent experiments using adult rat ventricular myocytes isolated from different rats. *P < 0.05 vs. control; †*P* < 0.05.

To determine which of the cardiac prohypertrophic signaling pathways are inhibited by NR4A2 overexpression in cardiomyocytes, ARVMs were stimulated for 10 min with isoproterenol, and phosphorylation of key effector proteins was investigated with Western blot. Phosphorylative activation of the transcription factor cAMP response element binding (CREB) protein at serine 133, a major event in the propagation of cAMP-PKA signaling to the nucleus, was not affected by NR4A2 overexpression, nor was the inhibitory phosphorylation of glycogen synthase kinase-3β (GSK-3β) at serine 9, a potent inhibitory mechanism of cardiac hypertrophy (Fig. 4, *A* and *B*). Although the phosphorylation status of the extracellularly regulated kinases (ERKs) 1 and 2 and of the Akt kinase remained unchanged under basal conditions, the activation of the two kinases was reduced by 50% and 36%, respectively, under isoproterenol stimulation (Fig. 4, *C* and *D*). Phosphorylative activation of p70S6K at threonine 389, a downstream effector of both ERK and Akt kinase and a key step in stimulation of protein synthesis under β-adrenoceptor stimulation, was also decreased in presence of NR4A2 overexpression.

#### NR4A2-mediated inhibition of ERK1/2 signaling is dependent on increased dual-specificity phosphatase activity.

**Fig. 5.**
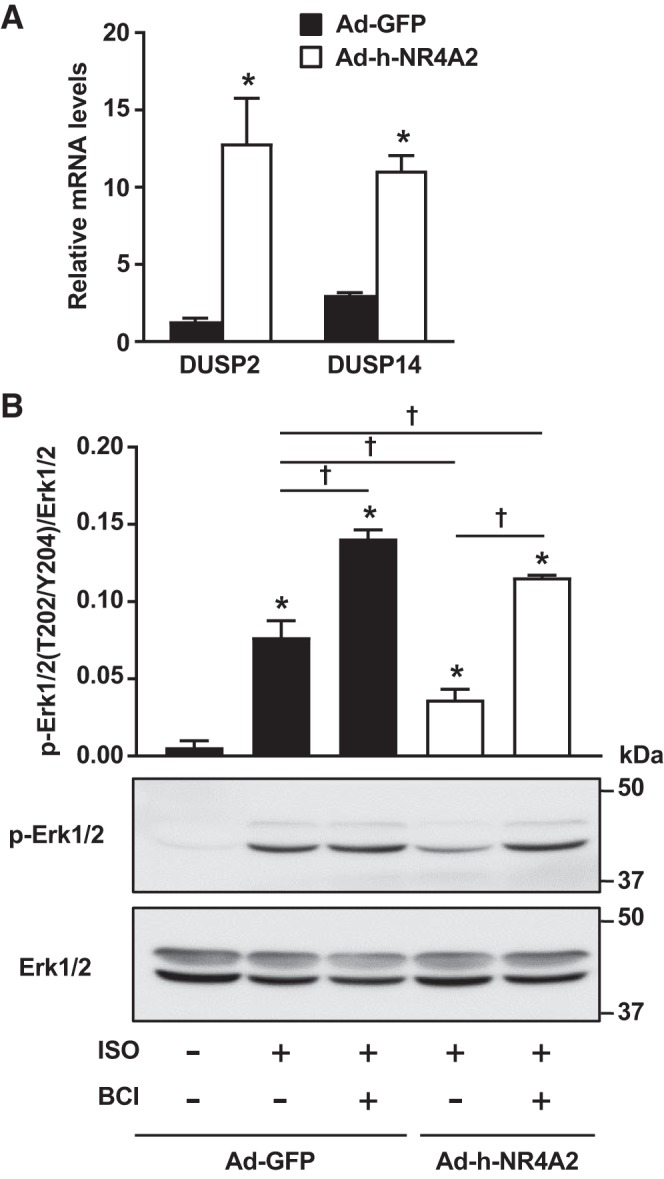
Inhibition of dual-specificity phosphatases (DUSPs) restores isoproterenol (ISO)-mediated activation of ERK signaling in nuclear receptor subfamily 4 group A (NR4A2)-overexpressing adult cardiomyocytes. *A*: real-time PCR quantification of DUSP2 and DUSP14 mRNA levels in adenovirus encoding green fluorescent protein (Ad-GFP)- and Ad-h-NR4A2-transduced cells. Pairwise comparison was performed with unpaired Student’s *t*-test. Data are means ± SE of 5 independent experiments using adult rat ventricular myocytes (ARVMs) isolated from different rats. *P < 0.05. *B*: GFP-expressing control and NR4A2-overexpressing cells were preincubated for 5 min with 5 µmol/L BCI [(E)-2-benzylidene-3-(cyclohexylamino)-2,3-dihydro-1*H*-inden-1-one] before stimulation for 10 min with 10 µmol/L ISO in presence of BCI. Phosphorylation (p) status of ERK1/2 was then quantified by immunoblotting. Interaction between multiple groups was determined by one-way ANOVA, including Newman-Keuls post hoc analysis when significant interaction occurred. Data are means ± SE of 3 independent experiments using ARVMs isolated from different rats. *P < 0.05 vs. control untreated Ad-GFP-expressing cells; †*P* < 0.05.

A closer analysis of the RNA sequencing data revealed that the dual-specificity phosphatases 2 (DUSP2/PAC-1) and 14 (DUSP14/MKP-6) were upregulated more than sevenfold following NR4A2 overexpression. The increase in DUSP2 and DUSP14 mRNA levels was confirmed by real-time PCR (Fig. 5*A*). Both DUSP2 and DUSP14 are established phosphatases for stress kinases ERK1/2, p38 MAP kinase and JNK (32). Moreover, DUSP2 is known for having a greater affinity for ERK and to localize in the nucleus, where it can counteract the effects of MAPK on gene transcription (15). To determine whether increased NR4A2 expression inhibits isoproterenol-mediated activation of ERK1/2 through DUSP-dependent dephosphorylation, ARVMs were pretreated with BCI, an allosteric inhibitor of DUSP, before stimulation with the β-adrenergic agonist. Pretreatment with BCI increased ERK1/2 stimulation in both control and NR4A2-overexpressing cells. However, the increase was comparatively more important in NR4A2-overexpressing cardiomyocytes: ERK1/2 phosphorylation increased more than threefold, reaching an activation level that was higher than the one detected in Ad-GFP expressing cells treated with isoproterenol only, but not significantly different from the same control cells pretreated with BCI (Fig. 5*B*). Thus, NR4A2-mediated inhibition of isoproterenol-dependent ERK signaling in ARVMs is caused by increased DUSP activity.

## DISCUSSION

The goals of this study were to determine the regulation of expression and subcellular localization of NR4A2 in adult cardiomyocytes subjected to isoproterenol treatment and to establish the consequences of such regulation for the physiological response of the cells to sustained β-adrenergic signaling stimulation. Isoproterenol induced expression and transient nuclear localization of NR4A2 in ARVMs. Chronic overexpression of NR4A2 via adenoviral transduction similarly led to increased nuclear localization of the transcription factor and induced significant transcriptional remodeling in the adrenoceptor, calcium, cell growth, and proliferation signaling pathways. Impaired growth response of NR4A2-overexpressing cardiomyocytes to chronic β-adrenergic stimulation coincided with upregulation of DUSP2 and DUSP14, two known inhibitors of mitogen-activated protein kinases (MAPK). At the cell signal transduction level, we found that NR4A2 inhibition of Akt and ERK signaling was linked to decreased p70S6K activation and to decreased rates of protein synthesis. We also demonstrated that ERK inhibition occurred through increased DUSP expression and activity.

Myocardial hypertrophy is a complex biological process that is activated to maintain cardiac workload in the face of environmental stress. By integrating the neurohumoral and biomechanical stimuli relayed through various intracellular signal transduction pathways, transcription factors play a critical role in determining rates of protein turnover and growth of cardiomyocytes (2, 17). While initially adaptive in the cardiac response to pathological stimuli, extensive growth of cardiac myocytes eventually contributes to the development of heart failure (14). Thus, a fine balance between activity of prohypertrophic and antihypertrophic factors is vital to the maintenance of proper cardiac function, and increased knowledge of the transcriptional control mechanisms in this area holds great promise for better management of heart failure (19).

In 2009, the Muscat team (31) reported that the mRNAs for all three members of the NR4A family of nuclear receptors are expressed in the mouse heart and are rapidly induced following isoproterenol treatment. In contrast with other nuclear receptors, activity of the NR4A members is ligand independent and mostly regulated at the level of gene expression (40). Although baseline expression of NR4A2 in the heart was the lowest among all three family members, its induction in response to β-adrenergic signaling was the greatest, with mRNA levels increasing 65-fold after 1 h of stimulation, well above all other organs and tissues analyzed (31). The time course and induction level of NR4A2 in the mouse heart are comparable to that observed here in isolated ARVMs, suggesting that myocytes are a major contributor to NR4A2 expression in the mammalian heart. Moreover, our findings of increased NR4A2 immunoreactivity in cardiomyocyte nuclei further supports a role for the nuclear receptor in transcriptional remodeling following β-adrenergic signaling activation.

Although the NR4A receptors have been implicated in the regulation of cell growth and proliferation in areas such as cancer and vascular biology (24, 34, 44), their impact on cardiac physiology has only started to be investigated recently. Initial studies from the Sussman group reported that NR4A1 translocates from the nucleus to the mitochondria in cardiomyocytes to initiate apoptosis following ischemia-reperfusion injury. The authors also reported marked NR4A1 immunoreactivity in cardiomyocyte nuclei following transaortic constriction in mice, a finding that already suggested at the time that NR4A1 participates in transcriptional reprogramming of adult cardiomyocytes in response to pressure overload (9). Two other independent studies combining in vitro analyses in cultured neonatal rat ventricular myocytes (NRVMs) with in vivo genetic manipulations in mice concluded that NR4A1 inhibits the cardiac hypertrophic response to β-adrenoceptor activation (27, 42). Conversely, NR4A3 was identified as a mediator of isoproterenol-induced cardiomyocyte growth through upregulation of poly(ADP-ribose) polymerase-1 activity (11). More recently, NR4A2 has been proposed to protect cardiomyocytes from myocardial infarction injury by promoting autophagy (21). To our knowledge, a role for NR4A2 in the regulation of cardiac myocyte growth had never been explored. In addition, the extent of the transcriptional remodeling events triggered by any of the NR4A members in cardiomyocytes remained unknown.

Because of their similarity in morphology and behavior with cardiomyocytes in intact tissue, ARVMs are particularly adapted to the investigation of the transcriptional and physiological effects of NR4A2 overexpression. Unlike NRVMs, ARVMs have a mature phenotype, which was maintained for the whole duration of the experiments (Supplemental Fig. S1). Cultures of NRVMs are frequently contaminated by other cell types (43), which would have complicated the analysis of the RNA sequencing data. In addition, ARVMs can be maintained in serum-free culture medium, thereby avoiding the confounding effects of growth factors in data interpretation (33). This was particularly important in our study, because several growth factor-activated signal transduction pathways were altered with increased NR4A2 expression (Table 1). All together, the RNA sequencing data clearly indicate that NR4A2 plays a significant role in structural remodeling of the cardiomyocyte.

Cardiac hypertrophy is characterized by an increase in cardiomyocyte size, enhanced protein synthesis, and reexpression of fetal genes such as BNP. As previously shown by others (10, 36–38), all those features were recapitulated in isolated cardiomyocytes subjected to chronic isoproterenol stimulation. Overexpression of NR4A2 was sufficient to reverse the prohypertrophic effects of isoproterenol on ARVMs. Although the RNA sequencing results indicated that NR4A2 might block cardiomyocyte growth induced by β-adrenoceptor stimulation through multiple mechanisms, regulators of the MAPK pathway including more than 10 members and regulators of the Ras superfamily of small guanosine triphosphatases, a member of the MAPKKK family, and several dual-specificity phosphatases were particularly represented (Table 1). This suggested that the antihypertrophic effects of NR4A2 are mediated, at least in part, though inhibition of the MAPK signaling pathway. This was confirmed by our finding of a reduction in ERK1/2 phosphorylation following isoproterenol treatment.

The MEK1-ERK1/2 pathway occupies a central regulatory position in the integration of both physiological and pathological stimuli promoting myocyte growth and cardiac hypertrophy (7). Both in vitro and in vivo data have demonstrated that ERK1/2 activation is required for cardiomyocyte growth and cardiac hypertrophy in response to β-adrenergic signaling. β-Adrenoceptors induce cardiac hypertrophy through canonical phosphorylation of ERK1/2 at Thr^202^/Tyr^204^ via Gα_s_ and Gβ_γ_-mediated autophosphorylation at Thr^188^ to increase rates of protein synthesis in cardiomyocytes (23, 38). Seminal work from the Molkentin group (35) revealed that calcineurin-nuclear factor of activated T cells (NFAT) and MEK1-ERK form a complex in the cytosol of cardiomyocytes to integrate both calcium and growth signals to coordinate nuclear gene expression and cardiac growth response. Besides transcriptional control of gene expression, the main mechanism by which ERK promotes growth of adult cardiomyocytes is through phosphorylation and activation of p70S6K, which enhances the efficiency of translation and, hence, the synthesis of protein during the hypertrophic response (18, 39).

Feedback inactivation of ERK1/2 is mediated mainly by phosphatases such as protein phosphatase-2A (PP2A) or dual-specificity phosphatases (DUSPs), which dephosphorylate either or both regulatory tyrosine and threonine residues in the activation loop (12). There are 61 DUSPs of heterogeneous form and function, which can be grouped on the basis of the presence of specific domains and sequence similarity. In cultured NRVMs, overexpression of DUSP1/MKP-1 is sufficient to partly inhibit the increase in cell surface area induced by phenylephrine treatment (13). Likewise, adenovirus-mediated overexpression of MKP-1 in NRVMs prevents cell growth in response to multiple prohypertrophic stimuli, while in vivo it reduces cardiac MAPK activation in response to phenylephrine and inhibits pressure overload- and catecholamines-induced hypertrophy in mice (6). Both the mitogen-activated protein kinase phosphatase (MKP) DUSP2/PAC-1 and the atypical DUSP14/MKP-6, which were upregulated with NR4A2 overexpression in ARVMs, have been shown to modulate ERK activity. Regarding DUSP15, whose expression was downregulated, specific substrates are currently unknown (32). Although BCI-mediated blockade of DUSP activity has only been reported for DUSP1 and DUSP6 so far (28), treatment of the NR4A2-overexpressing ARVMs with the small molecular inhibitor efficiently restored activation of ERK in response to isoproterenol. The results suggest that increased DUSP activity is one of the mechanisms by which NR4A2 antagonizes β-adrenergic-mediated protein synthesis and growth in adult cardiomyocytes.

In conclusion, we have shown that activation of β-adrenergic receptors induces expression and nuclear localization of the nuclear receptor NR4A2 in adult cardiomyocytes. Increased NR4A2 immunoreactivity in cardiomyocyte nuclei is sufficient to induce a genomewide transcriptional remodeling with differential regulation of more than 850 genes. Based on our in vitro signal transduction pathways analyses, NR4A2 may inhibit the growth response of cardiomyocytes to isoproterenol in part by DUSP-mediated blockade or ERK signaling and inhibition of protein synthesis. We acknowledge that our study is only uncovering a small portion of the molecular effects of NR4A2 on cardiomyocyte physiology. Thus, inhibition of PI3K-Akt signaling and of other stress-related kinases, p38 and JNK, is also likely to contribute to the observed phenotype. In addition, this study did not address whether the differentially regulated genes were directly or indirectly targeted by NR4A2. Future studies will aim to address these remaining questions and will determine whether the findings can be transposed in vivo in an animal model of pathological hypertrophy.

## GRANTS

This work was supported by Grants R00 HL-112952, R01 HL-136438, and P01 HL-051971 from the National Heart, Lung, and Blood Institute. Work performed through the University of Mississippi Medical Center Molecular and Genomics and Imaging Core Facilities was supported, in part, by funds from the National Institute of General Medical Sciences, including Mississippi INBRE (P20 GM-103476), Obesity, Cardiorenal and Metabolic Diseases-COBRE (P20 GM-104357), Mississippi Center of Excellence in Perinatal Research (MS-CEPR)-COBRE (P20 GM-121334), and Center for Psychiatric Neuroscience-COBRE (P30 GM-103328). The content of the paper is solely the responsibility of the authors and does not necessarily represent the official views of the National Institutes of Health.

## DISCLOSURES

No conflicts of interest, financial or otherwise, are declared by the authors.

## AUTHOR CONTRIBUTIONS

S.A. and R.H. conceived and designed research; S.A. and Y.K.H. performed experiments; S.A., Y.K.H., and R.H. analyzed data; S.A., Y.K.H., and R.H. interpreted results of experiments; S.A. and R.H. prepared figures; S.A. drafted manuscript; S.A. and R.H. edited and revised manuscript; S.A. and R.H. approved final version of manuscript.
